# Adolescent gut microbiome imbalance and its association with immune response in inflammatory bowel diseases and obesity

**DOI:** 10.1186/s12866-024-03425-y

**Published:** 2024-07-19

**Authors:** Minjae Joo, Seungyoon Nam

**Affiliations:** 1 Department of Health Sciences and Technology, Gachon Advanced Institute for Health Sciences and Technology (GAIHST), Gachon University, Incheon, 21999 Korea; 2 Department of Genome Medicine and Science, AI Convergence Center for Medical Science, Gachon Institute of Genome Medicine and Science, Gachon University Gil Medical Center, Gachon University College of Medicine, Dokjeom-Ro 3Beon-Gil, 38-13, Namdong-Gu, Incheon, 21565 Republic of Korea

**Keywords:** Adolescents, Microbiome, Obesity, Inflammatory bowel diseases

## Abstract

**Background:**

Recently, there has been an increase in the number of studies focusing on the association between the gut microbiome and obesity or inflammatory diseases, especially in adults. However, there is a lack of studies investigating the association between gut microbiome and gastrointestinal (GI) diseases in adolescents.

**Method:**

We obtained 16S rRNA-seq datasets for gut microbiome analysis from 202 adolescents, comprising ulcerative colitis (UC), Crohn’s disease (CD), obesity (Ob), and healthy controls (HC). We utilized Quantitative Insights Into Microbial Ecology (QIIME) and Phylogenetic Investigation of Communities by Reconstruction of Unobserved States (PICRUSt) to acquire Operational Taxonomic Units (OTUs). Subsequently, we analyzed Kyoto Encyclopedia of Genes and Genomes (KEGG) Orthology (KO) terms and pathway enrichment for the identified OTUs.

**Results:**

In this study, we investigated the difference between the gut microbiomes in adolescents with GI diseases and those in healthy adolescents using 202 samples of 16S rRNA sequencing data. The distribution of the six main gut microbiota (i.e., unclassified *Dorea*, unclassified *Lachnospiraceae*, unclassified *Ruminococcus*, *Faecalibacterium prausnitzii*, *Prevotella copri*, unclassified *Sutterella*) was different based on the status of obesity and inflammatory diseases. Dysbiosis was observed within *Lachnospiraceae* in adolescents with inflammatory diseases (i.e., UC and CD), and in adolescents with obesity within *Prevotella* and *Sutterella*. More specifically, our results showed that the relative abundance of *Faecalibacterium prausnitzii* and unclassified *Lachnospiraceae* was more than 10% and 8% higher, respectively, in the UC group compared to the CD, Ob, and HC groups. Additionally, the Ob group had over 20% and over 3% higher levels of *Prevotella copri* and unclassified *Sutterella*, respectively, compared to the UC, CD, and HC groups. Also, inspecting associations between the six specific microbiota and KO terms, we found that the six microbiota -relating KO terms were associated with NOD-like receptor signaling. These six taxa differences may affect the immune system and inflammatory response by affecting NOD-like receptor signaling in the host during critical adolescence.

**Conclusion:**

In this study, we discovered that dysbiosis of the microbial community had varying degrees of influence on the inflammatory and immune response pathways in adolescents with inflammatory diseases and obesity.

**Supplementary Information:**

The online version contains supplementary material available at 10.1186/s12866-024-03425-y.

## Background

Microorganisms coexist in various parts of the body, including the skin, oral cavity, and intestines [1]. Only recently, the reasons for this coexistence are beginning to be understood, although they largely remain unclear. Among all the symbiotic microorganisms, the Human Microbiome Project [2] was initiated to investigate and understand the group of microorganisms associated with human health and diseases. Thereafter, several studies were conducted and it was found that gut microbiome plays a significant role in the mechanisms of inflammation, immunity, and diseases [3–5]. Understanding the role of the gut microbiome is becoming increasingly important for understanding various mechanisms of the human body.

Inflammation is a biological response of the immune system to various factors. NF-kB, MAPK, and JAK-STAT are the three main signaling pathways essential for inflammation. Impaired regulation of these pathways can lead to diseases associated with inflammation [6]. Chronic inflammation is associated with both infectious and non-infectious diseases [7]. The association between the gut microbiome and inflammatory diseases in adults is well-known [8]. Inflammatory Bowel Disease (IBD), including Crohn’s disease (CD) and ulcerative colitis (UC) are the representative inflammatory diseases associated with inflammatory responses, and there is active research on their association with the gut microbiome [9, 10]. Obesity is associated with inflammatory responses, and research has focused on the gut microbiome in individuals with obesity [11]. IBD is a disorder that has seen a consistent increase in incidence worldwide, with an annual percentage change ranging from 1.2% to 23.3% from the 1930s to 2010 [12]. The prevalence of obesity in 2015 was 12% among adults, having increased since the 1980s, and is a disease that shows a prevalence rate more than double in over 70 countries [13]. The composition of the gut microbiome differs between adults and adolescents [14]. Recent studies on adults have reported that changes in the gut microbiome are associated with the onset of IBD and obesity in adults [15]. Specifically, an increase in *Lachnospiraceae* has been observed in adult IBD patients [16], while an increase in *Firmicutes* has been linked to obesity [17]. Given the established findings between the gut microbiome, inflammation, IBD, and obesity in adults, we hypothesize that microbial community changes related to IBD and obesity may exist in adolescents. However, there has been a lack of research focusing on this age group and this study aims to fill that gap.

In this study, we examined the taxonomic classification and the composition (proportion) of the gut microbiota during adolescence, a period of active physiological changes. We found considerable differences between healthy individuals and those with inflammatory diseases. We collected and analyzed 16S rRNA-seq data from adolescents in the healthy control (HC, *n* = 92), obese (Ob, *n* = 68), CD (*n* = 37), and UC (*n* = 5) groups. Again, we hypothesize that there will be significant differences in the gut microbiome between healthy adolescents and those having obesity and IBD due to the unique physiological and immunological characteristics of this age group.

## Results

### Diversity analysis: sample analysis from adolescent fecal specimens using 16S rRNA-seq data (Analysis of gut microbial communities of adolescents in HC, Ob and IBD)

**Fig. 1:**
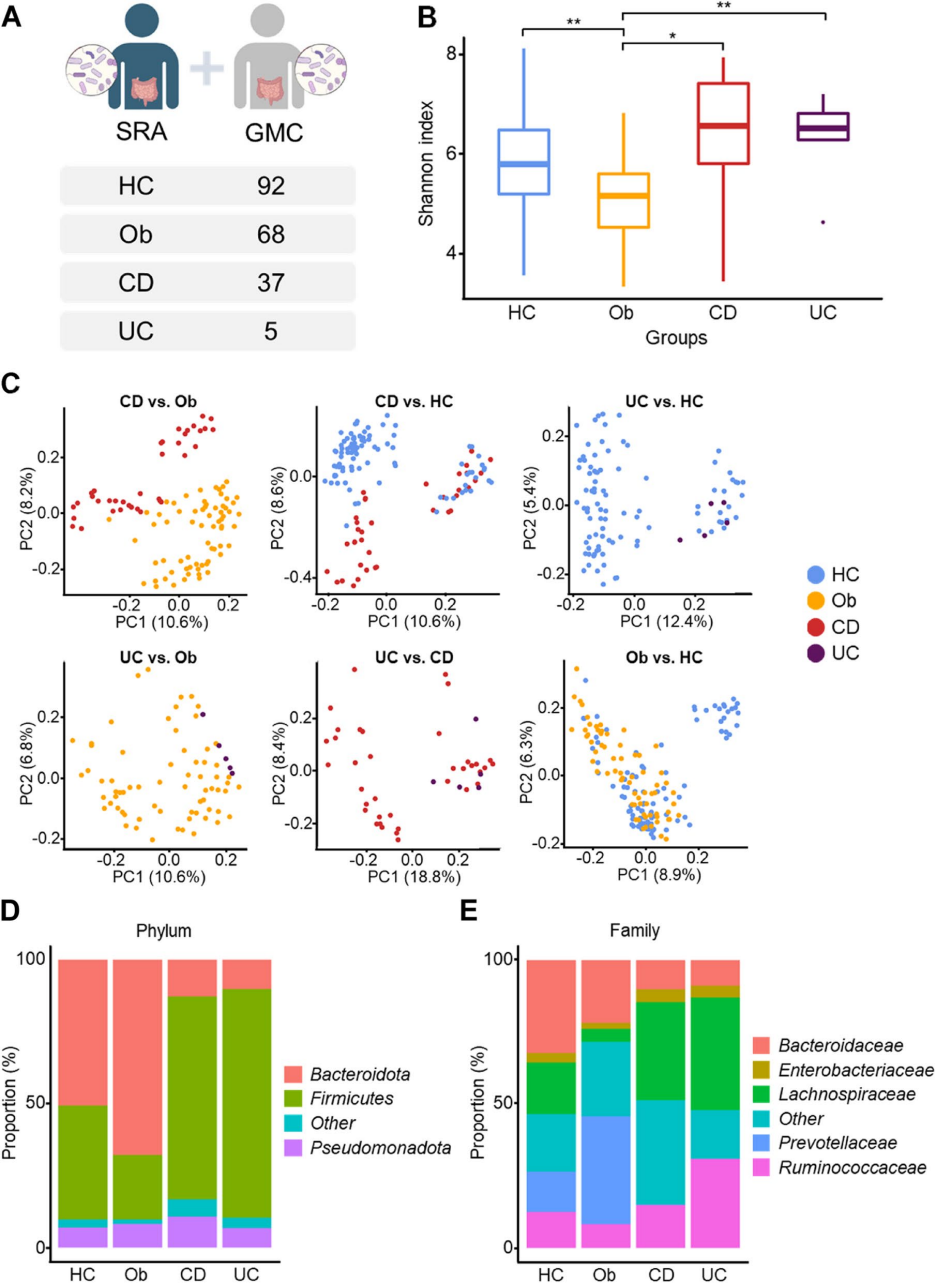
Overview of the dataset composition and distribution of gut microbiome by group. We analyzed the abundance and diversity of gut microbiome from the collected data using QIIME. **A** Dataset composition and the number of samples used in the analysis. Based on the collected data, we classified the patients into Healthy Control (HC), obese (Ob), Crohn’s disease (CD), ulcerative colitis (UC) groups. **B** Analysis of gut microbiome abundance by group. Boxplot from alpha-diversity of the group (x-axis) vs. Shannon index (y-axis), showing abundance. ***, *p* < 0.001; **, *p* < 0.01; * *p* < 0.05. **C** PCoA plots for each group (CD vs. Ob, CD vs. HC, UC vs. HC, UC vs. Ob, UC vs. CD, Ob vs. HC). **D**-**E** Gut microbiota diversity at the Phylum (**D**) and Family (**E**) levels is shown in a barplot. They were integrated into ‘Other’ at the Phylum level, with the exception of the majority of taxa, and into ‘Other’ at the Family level for taxa that did not belong to the corresponding Phylum level

A total of 202 samples were collected for analysis of the gut microbial community in adolescents, across healthy controls (HC), obese (Ob), and inflammatory bowel disease (IBD) groups (i.e., Crohn’s Disease—CD and Ulcerative Colitis—UC) (Fig. 1A).

To assess the diversity of the gut microbial community in each group, we calculated alpha diversity using Shannon metrics on Quantitative Insights Into Microbial Ecology (QIIME) [18]. No significant difference was observed in UC vs. CD, UC vs. HC, and CD vs. HC. However, significant differences were observed between UC and Ob, CD and Ob, and Ob and HC groups (*P*-value = 0.048, 0.006, and 0.006, respectively) (Fig. 1B).

To examine the similarities and differences among the groups, beta diversity was presented using 2-Dimensional Principal Coordinate Analysis (2D PCoA) (Fig. 1C and Supplementary Figure S1). Of interest, the 2D PcoA in CD vs. Ob showed a distinct separation (the upper left panel in Fig. 1C).

### Taxa proportion analysis at the levels of phylum and family: sample analysis from adolescent fecal specimens using 16S rRNA-seq data (analysis of gut microbial communities of adolescents in HC, Ob and IBD)

To determine differences in the gut microbial community among groups, we examined the proportions of taxa at the phylum level. *Firmicutes* accounted for more than 70% of the proportion of the entire taxa in the CD and UC group, followed by *Bacteroidota* (synonym *Bacteriodetes*) and *Pseudomonadota*. *Bacteroidota* accounted for more than 50% of the proportion of the entire taxa in the HC and Ob group, followed by *Firmicutes* and *Pseudomonadota*. *Firmicutes* accounted for 79.5% and 70.5% of total taxa in UC and CD respectively, significantly higher than 22.4% and 39.6% observed in Ob and HC respectively. In contrast, the proportion of *Bacteroidota* in UC, CD, Ob, and HC was 10.2%, 12.8%, 67.8%, and 50.7%, respectively, showing a relatively low proportion in the IBD group (UC and CD). The proportion of *Pseudomonadota* in UC, CD, Ob, and HC was 6.8% 10.7%, 8.2%, and 6.9%, respectively, and no significant difference was observed among the groups (Fig. 1D).

*Lachnospiraceae* (a family of *Firmicutes*) accounted for 39.1% and 34.1% of taxa in UC and CD, respectively, significantly higher than the proportions observed in the Ob (4.5%) and HC (17.9%) groups. The proportion of *Bacteroidaceae* (belonging to *Bacteroidota*) in UC and CD was 8.9% and 10.2%, respectively; which was lower than that in the Ob (21.8%) and HC (32.3%) groups. The proportion of *Prevotellaceae* in UC and CD was 0.03% and 0.2%, respectively; which was lower than that in HC (14%) and Ob (37.4%). The proportion of *Ruminococcaceae* (belonging to Firmicutes) in UC was 30.9%; which was higher than that in the CD (14.9%), Ob (8.2%), and HC (12.5%) groups (Fig. 1E).

Regarding our hypothesis, we observed that adolescents in the IBD group had higher levels of *Lachnospiraceae* and lower levels of *Bacteroidaceae* and *Prevotellaceae* compared to healthy adolescents (Fig. 1E). Specifically, in UC group had higher levels of *Ruminococcaceae* compared the other group (Fig. 1E). Also, adolescents in the obesity group exhibited higher levels of *Prevotellaceae* and lower levels of *Lachnospiraceae* compared to the healthy adolescent group (Fig. 1E).

### Taxa proportion analysis at the level of genus: UC group had a high proportion of *Faecalibacterium prausnitzii* and unclassified *Lachnospiraceae*; Ob group had a high proportion of *Prevotella copri* and unclassified *Sutterella*

**Fig. 2:**
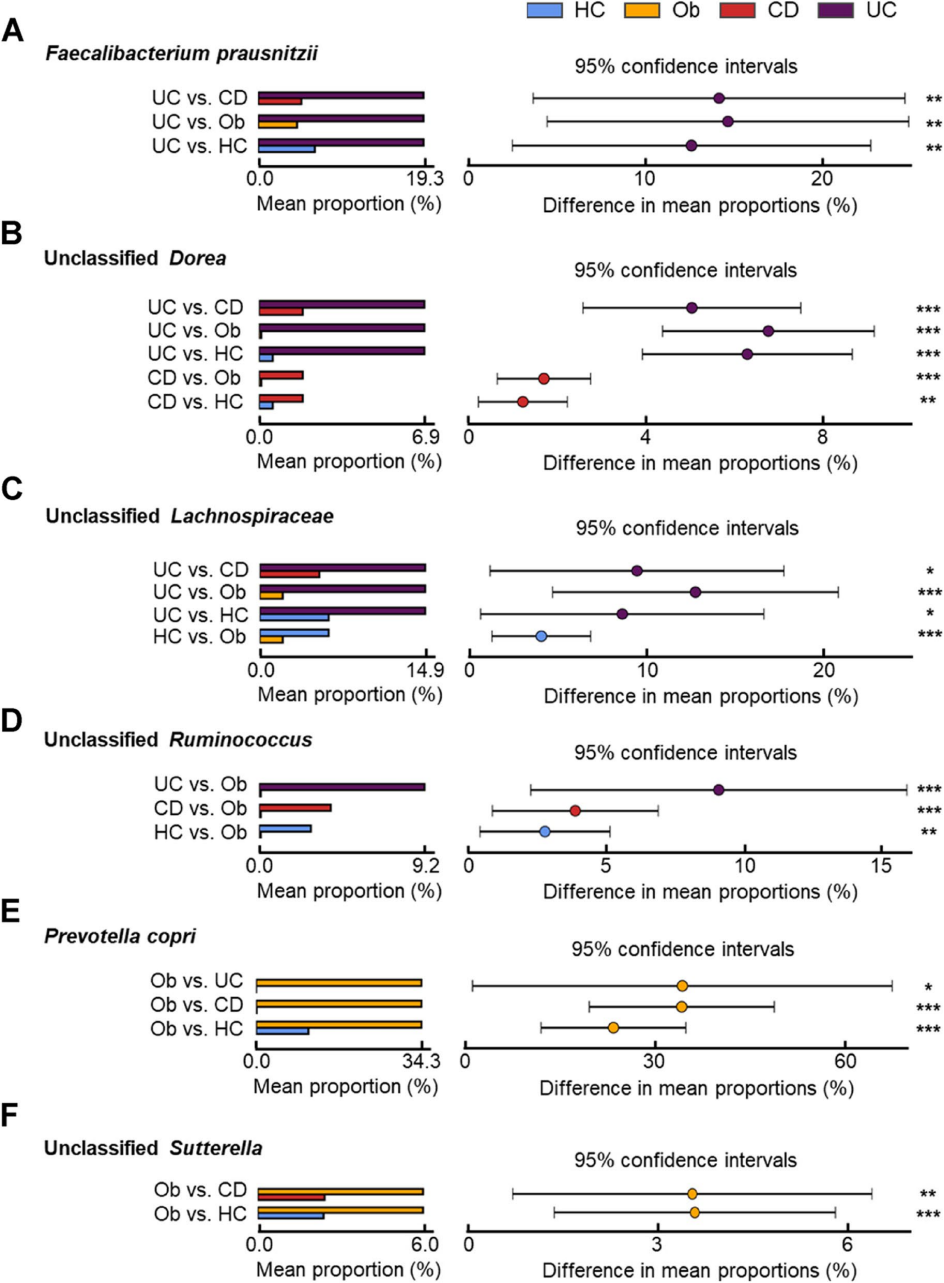
Six representative taxa show significant differences based on obesity and disease status. We present the mean proportion of the respective taxa in each group and the difference in mean proportion when comparing the groups. **A***Faecalibacterium prausnitzii.***B** Unclassified *Dorea.***C** Unclassified *Lachnospiraceae.***D** Unclassified *Ruminococcus.***E***Prevotella copri.***F** Unclassified *Sutterella*. ***, *p* < 0.001; **, *p* < 0.01; * *p* < 0.05

In the family level taxa (*Lachnospiraceae*, *Bacteroidaceae*, *Prevotellaceae*) that showed differences in the previous section (Fig. 1E), to identify which taxa at the level of genus/species varied among the groups, we used Operational Taxonomy Units (OTUs) data from QIIME to conduct Phylogenetic Investigation of Communities by Reconstruction of Unobserved States (PICRUSt) [19] and STatistical Analyses of Metagenomic Profiles (STAMP) [20] analyses. At the genus level, 55 taxa showed significant differences among the groups (FDR q-value < 0.05) (Supplementary Table S1). These 55 taxa mainly corresponded to *Lachnospiraceae*, *Ruminococcaceae*, *Prevotellaceae*, and *Enterobacteriaceae* at the family level (Fig. 1E). Of the 55 taxa that showed statistically significant (Benjamini–Hochberg FDR < 0.05) different proportions among groups, six taxa (*Faecalibacterium prausnitzii*, unclassified *Dorea*, unclassified *Lachnospiraceae*, unclassified *Ruminococcus*, *Prevotella copri*, and unclassified *Sutterella*) (Fig. 2) showed a mean proportion of ≥ 5% in the group with the highest proportion.

UC group had a significantly higher abundance (19.3%) of *Faecalibacterium prausnitzii*, a member of the *Ruminococcaceae* family, compared to other groups (5.05%, 4.55%, and 6.61% for CD, Ob, and HC, respectively) (Fig. 2A). UC group had a significantly higher abundance (6.88%) of unclassified *Dorea* than the other groups (1.82%, 0.09%, and 0.57% for CD, Ob, and HC, respectively), and the proportion in the CD group was significantly higher than that in the HC and Ob group (Fig. 2B). Among other *Dorea* species, the proportion of *Dorea formicigenerans* was significantly higher (1.18%) in the UC group compared to that in the other groups (0.49%, 0.01%, and 0.16% for CD, Ob, and HC, respectively), and its proportion in the CD group was significantly higher than that in the HC and Ob group (Supplementary Figure S2A). The proportion of *Dorea longicatena* was significantly higher (0.14%) in the UC group compared to that in the other groups (0.02%, 0%, and 0.01% for CD, Ob, and HC, respectively), and its proportion in the CD group was significantly higher than that in the Ob group (Supplementary Figure S2B).

The proportion of unclassified *Lachnospiraceae* was significantly higher in the UC group (14.89%) than that in the other groups (5.37%, 2.07%, and 6.2% for CD, Ob, and HC, respectively), and its proportion in the HC group was significantly higher than that in the Ob group (Fig. 2C). The proportion of unclassified *Ruminococcus* was in the Ob group (0.09%) was significantly lower than that in the other groups (9.16%, 3.96%, and 2.86% for UC, CD, and HC, respectively) (Fig. 2D).

The proportion of *Prevotella copri* was significantly higher in the Ob group (34.34%) than that in the other groups (0.005%, 0.1%, and 10.87% for UC, CD, and HC, respectively), and its proportion in the IBD group (UC and CD) was almost nil (Fig. 2E). Similarly, the proportion of unclassified *Sutterella*, was significantly higher in the Ob group (5.98%) than that in the CD (2.42%) and HC (2.38%) groups (Fig. 2F). Interestingly, the taxon belongs to Pseudomonadota, which did not show any differences at the phylum level (Fig. 1D).

Taxa including unclassified *Dorea*, unclassified *Lachnospiraceae*, unclassified *Ruminococcus*, *Dorea formicigenerans*, and *Dorea longicatena*, which all belong to the *Lachnospiraceae* family (Fig. 2B-D and Supplementary Figure S2), exhibited significantly higher proportions in the IBD group (UC and CD) compared to Ob and HC groups.

We observed gut microbiota diversity differences across groups, with significant variations in taxa proportions. Notably, the UC group had a statistically significantly higher distribution of *Faecalibacterium prausnitzii* by more than 10% (Fig. 2A) and unclassified *Lachnospiraceae* by more than 8% compared to the other groups (Fig. 2C), while the Ob group had a statistically significantly higher distribution of *Prevotella copri* by more than 20% (Fig. 2E) and unclassified *Sutterella* by more than 3% (Fig. 2F).

### Functional implications: relationship between ‘Kyoto Encyclopedia of Genes and Genomes (KEGG) Orthology terms’ (KO terms) and pathways with the taxa showing significantly different proportions

**Fig. 3:**
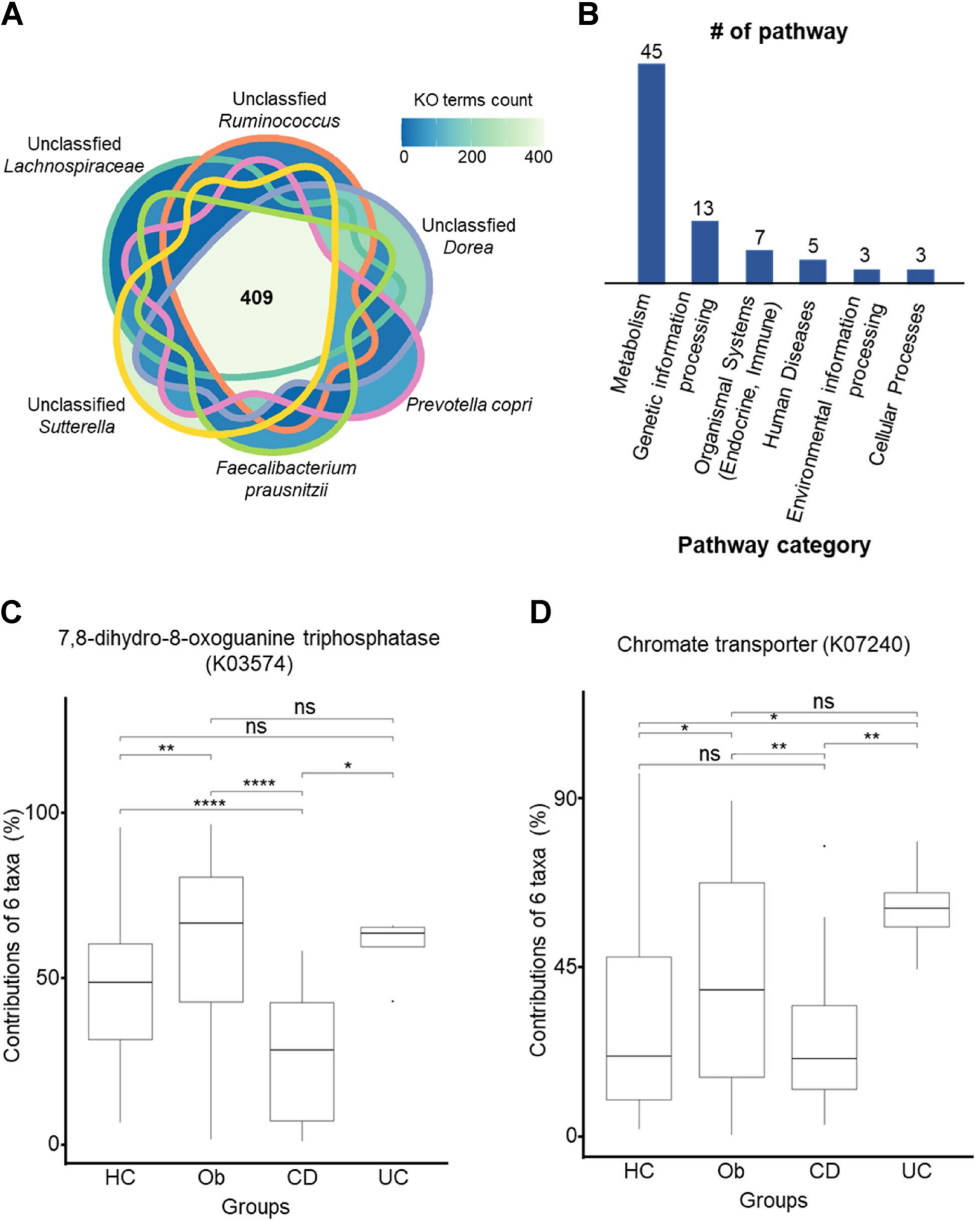
Analysis of KEGG orthology (KO) and pathways associated with six taxa. **A** 409 results from overlapping the KO terms that are associated with 6 representative taxa showing differences. **B** The number of pathways per category from among the 76 pathways. **C** 7,8-dihydro-8-oxoguanine triphosphatase (K03574). **D** Chromate transporter (K07240)

To identify the functions of the six genus/species-level taxa discovered through QIIME in the previous section, we used PICRUSt to investigate common features (i.e., KO terms) that showed large differences in proportions across the six taxa. We then mapped these functions to their respective pathways. A total of 409 KO terms were common (Fig. 3A), and 76 pathways were identified by mapping these KO terms onto the pathways (Fig. 3B and Supplementary Table S2). Of the categories Metabolism, Genetic information processing, Organismal Systems, Human Diseases, Environmental Information Processing, and Cellular Processes, more than half of the detected pathways (45/76) were related to Metabolism (Fig. 3B). Various effects of the gut microbiome on the body were found by mapping pathways of other categories.

**Fig. 4:**
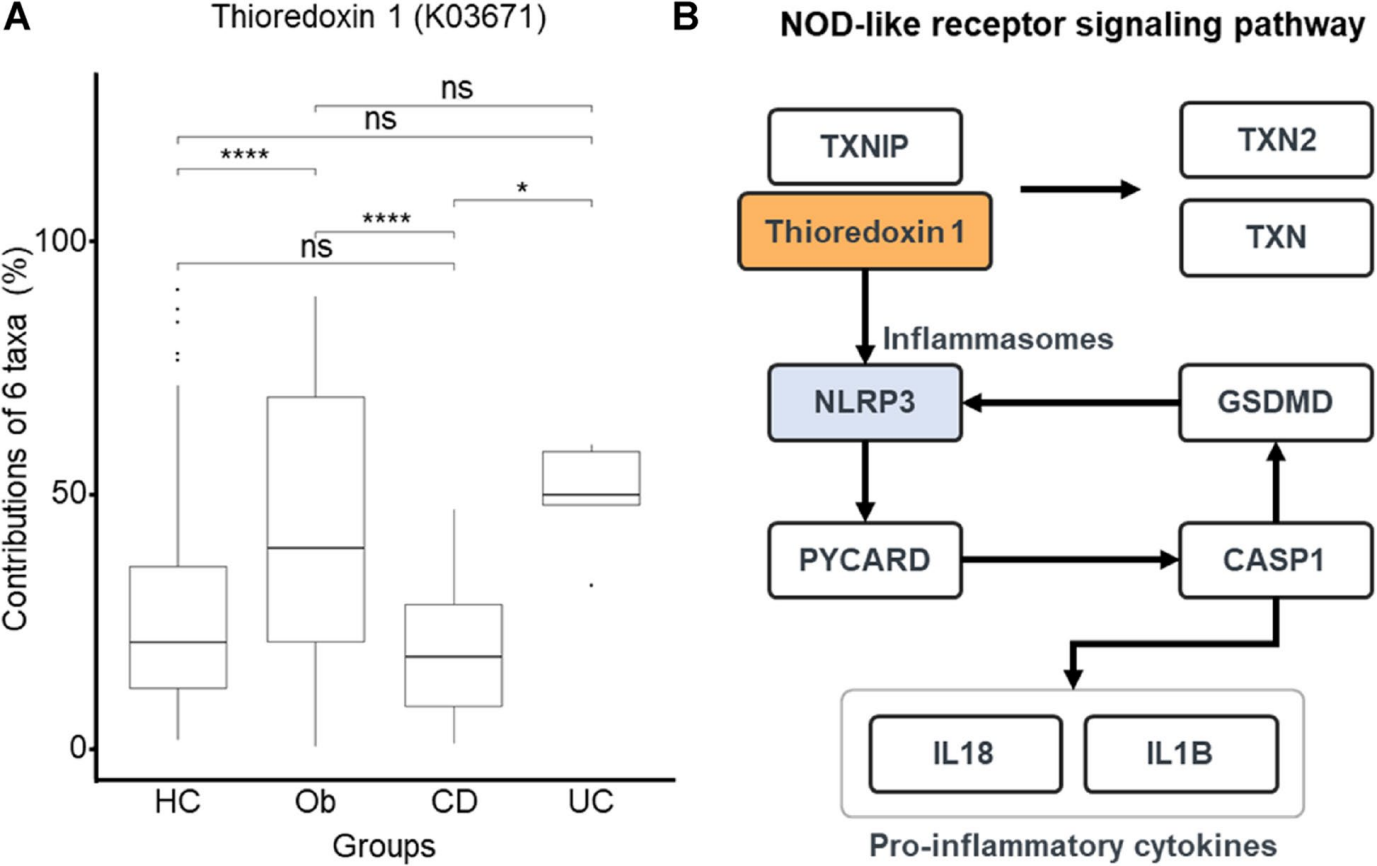
Differences in the contribution of thioredoxin 1 among groups and the analysis of the associated pathway. **A** Comparison of results among groups for pathways involved in the immune system category and the associated KO (thioredoxin 1, K03671). **B** Diagram representing the role of thioredoxin 1 (K03671) in NOD-like receptor signaling pathway

We determined the contribution of the six taxa to the 409 KO terms and investigated if these contributions significantly varied among the groups. We performed six pair-wise comparisons (i.e., UC vs. CD, UC vs. Ob, UC vs. HC, CD vs. Ob, CD vs. HC, and Ob vs. HC) using the Tukey Honestly Significant Difference (Tukey HSD) method. Of the six pair-wise comparisons, KO terms showing significant differences in four pair-wise comparisons were ‘7,8-dihydro-8-oxoguanine triphosphatase (K03574)’ (UC vs. CD, CD vs. Ob, CD vs. HC, Ob vs. HC) and ‘chromate transporter (K07240)’ (UC vs. CD, UC vs. HC, CD vs. Ob, Ob vs. HC) (Fig. 3C and D). KO term ‘thioredoxin 1 (K03671)’ showed significant differences in the three pair-wise comparisons (UC vs. CD, CD vs. Ob, and Ob vs. HC) (Fig. 4A). The KEGG database was queried and the NOD-like receptor signaling pathway was determined to be associated with ‘thioredoxin 1 (K03671)’ (Fig. 4B). We confirmed that microbial community dysbiosis in gut microbiota, which play various roles in the human body, occurs due to the incidence of diseases, and has a close relationship with inflammation [4]. Pathways crucial during adolescence, particularly those related to the immune system and inflammation, were also affected.

## Discussion

In this study, we sought to elucidate the variations in gut microbial composition among adolescents (10–19 years old) [21] in HC, Ob, and IBD states by using16S rRNA-seq datasets. We revealed the associations of the six taxa (i.e., unclassified *Dorea*, unclassified *Lachnospiraceae*, unclassified *Ruminococcus*, *Faecalibacterium prausnitzii*, *Prevotella copri*, and unclassified *Sutterella*) with IBD in adolescents, and the taxa have not been established yet in adolescent with IBD. Our novelty in this study is to add evidence of these six taxa having associations with IBD.

Expanding upon these associations quantitatively, our findings demonstrate significant variations in gut microbiota composition among different disease groups. The UC group exhibits over 10% and 8% higher relative abundances of *Faecalibacterium prausnitzii* (Fig. 2A) and unclassified *Lachnospiraceae* (Fig. 2C), respectively, in comparison to the CD, Ob, and HC groups. Conversely, the Ob group shows more than 20% higher levels of *Prevotella copri* (Fig. 2E) and over 3% higher levels of unclassified *Sutterella* (Fig. 2F) compared to the CD and HC groups.

Interestingly, our findings align with previous research conducted on adults where it was found that UC patients had higher proportions of *Firmicutes* but lower *Bacteroidota* when compared to control subjects [22]. Similarly, another study reported an elevated presence of *Faecalibacterium prausnitzii* in adult UC patients [23]. Adolescence involves significant physical, metabolic, and cognitive changes, primarily driven by hormonal shifts during puberty [24–27]. These hormonal fluctuations not only impact growth and body composition but also influence the gut microbiota [28, 29]. Our study aligns with recent research showing the maturation of the gut microbiota during adolescence, resembling that of adults, a process influenced by puberty-related hormonal changes [30, 31]. Prolonged disruptions in the gut microbiota during this crucial period may impede its ability to maintain host balance [32, 33]. In our findings, we observed dysbiosis in six taxa in adolescents with UC, notably identifying an increased presence of *Faecalibacterium prausnitzii*, shared between adolescent and adult UC. This suggests a potential continuity of dysbiosis from adolescence into adulthood, potentially impacting UC development.

However, our results deviate from adult studies reporting decreased taxa belonging to *Lachnospiraceae* and *Ruminococcaceae* in adult CD patients along with depletion of *Dorea formicigenerans* within adult IBD patient groups when compared to adult non-IBD controls [34] as seen contrary from our findings in adolescents (Fig. 1E and Supplementary Figure S2). This contrast might be attributed to hormonal transition between adolescence and adulthood. In fact, Clinical research has identified correlations between estrogen and testosterone levels and changes in the composition and diversity of gut microbiota [28, 29]. Moreover, studies indicate that gut microbial communities can metabolize and alter sex hormones in ways that could significantly affect host physiology [28, 29]. This suggests a reciprocal relationship between the gut microbial communities and the hormones, which could be integral to adolescent growth and maturation [27]. This interplay could be pivotal for healthy adolescent growth, potentially mediated by gut microbiota-derived metabolites [27]. However, further investigation is required to address how these metabolites and sex hormones interact differentially between adolescent and adult IBD patients.

We searched for relevant publications for the six taxa (*Faecalibacterium prausnitzii*, unclassified *Dorea*, unclassified *Lachnospiraceae*, unclassified *Ruminococcus*, *Prevotella copri*, unclassified *Sutterella*) involved in inflammatory or immune responses. A greater abundance of *Faecalibacterium prausnitzii* in patients with inflammatory disease resulted in a greater response to anti-TNF-alpha treatment [35]. *Dorea* is closely related to immunoglobulin G (IgG) of the humoral immune response [36], and is vulnerable to antibiotics [37]. *Lachnospiraceae* produces short-chain fatty acids (SCFAs), and is gut-related diseases [38]. It can cause colitis, as suggested by a study on a mouse model [39]. *Ruminococcus,* belonging to *Lachnospiraceae,* produces SCFAs [40]. *Prevotella copri* is commonly found in the non-Western population who generally have a healthy diet [41]. *Sutterella* is generally found in patients with UC and is associated with inflammatory cytokine responses of the host [42]. It is known for its ability to break down the immunoglobulin A (IgA) of the immune system [43]. Overall, results from other studies corroborate our findings that these six taxa play a significant role in the inflammatory or immune response.

Also, we investigated publications comparing gut microbial communities between adults and adolescents with IBD within the same ethnicity or nationality. However, we could not find any such studies as far as we know. Additionally, a study on Mexican adolescents compared the gut microbiome of obese and normal-weight individuals, revealing a higher prevalence of *Prevotella* in obese adolescents compared to their normal-weight counterparts [44]. Another study comparing adolescents with new-onset CD to healthy controls found that microbes belonging to the *Ruminococcaceae* family were more prevalent in the new-onset CD group [45]. These studies are aligned with our findings.

Our findings in Fig. 4 suggest that KO terms relating to the six identified taxa (unclassified *Dorea*, unclassified *Lachnospiraceae*, unclassified *Ruminococcus*, *Faecalibacterium prausnitzii*, *Prevotella copri*, unclassified *Sutterella*) are associated with thioredoxin 1, which is closely linked with NLRP3 [46, 47]. Since both thioredoxin 1 and NLRP3 are components of the NOD-like receptor signaling pathway [46, 47], we propose that these six taxa are likely involved in modulating inflammation and immune responses. Specifically, in the host, thioredoxin 1 enhances the NLRP3 inflammasome, thereby promoting inflammation [48]. The NOD-like receptor signaling pathway, which includes the genes *IFNG*, *GBP5*, and *NLRP3*, is crucial for regulating immune responses in patients with UC and CD [49]. The gut microbiota provides the host with unique pathogen-associated molecular patterns (PAMPs) and damage-associated molecular patterns (DAMPs) that trigger innate immune responses [50, 51]. The NLRP3 inflammasome recognizes and responds to PAMPs and DAMPs through pattern recognition receptors (PRRs) [52, 53]. This interaction allows the NLRP3 inflammasome to influence immunity and metabolism, regulating the production of IL-1β and IL-18 [54, 55]. Activated IL-1β, in turn, promotes inflammation [55]. In patients with IBD, the disruption of gut microbial balance induced the activation of the NLRP3 inflammasome in intestinal macrophages [56, 57]. The activation of the NLRP3 inflammasome results in elevated levels of inflammatory cytokines, thereby exacerbating inflammatory responses in the gut [56, 57]. Taken together, our findings indicate that the alterations in the relative abundance of the six taxa may modulate the NOD-like receptor signaling pathway, thereby influencing the immune system and inflammatory responses in adolescents, a critical period for gut microbiome development and immune maturation.

Of the 76 significant pathways, 45 were related to the metabolism of lipids, carbohydrates, energy, nucleotides, amino acids, cofactors, and vitamins. Various lipid species, including fatty acids and their metabolites, sterols, complex lipids (e.g., glycerophospholipids and sphingolipids), and lipoproteins, possess immunomodulatory and pro-/anti-inflammatory properties [58]. Four lipid metabolism pathways (fatty acid biosynthesis, fatty acid metabolism, glycerolipid metabolism, and glycerophospholipid metabolism) were observed, confirming their role in the inflammatory and immune response (Supplementary Table S2). Riboflavin and thiamine metabolisms affect inflammatory responses by decreasing TNF-alpha and IL-6 production [59]. The results from our study also included the riboflavin and thiamine metabolisms. Oxidative phosphorylation, which is involved in metabolism for the immune cells to function during inflammation, was also identified [60]. This result is consistent with other studies on gnotobiotic mouse models or human fecal samples [61, 62].

We discuss the implications of the differences observed in the six taxa for therapeutic potential in IBD, a prominent chronic condition. Given the dysbiosis of the six taxa identified in our findings among adolescents, there is a need for intervention. Fecal microbiota transplantation (FMT) presents a potential solution, entailing the transfer of fecal material rich in beneficial microbes from a healthy donor to a recipient experiencing dysbiosis, thereby fostering a favorable shift in the recipient’s gut microbiome composition [63, 64].

Despite not being able to track whether the analyzed samples were exposed to inflammation and other diseases after adulthood, this study provides crucial insights into the impact of diversity and taxonomic composition on the adolescent gut microbial community. It also sheds light on potential disease risks during adolescence. Our study may have certain biases due to the small sample size of UC (*n* = 5) compared to the other groups. Therefore, when interpreting the analysis of group-specific differences in gut microbiota, these biases should be taken into consideration. To mitigate these biases, future research is needed in a larger adolescent population with quantitative statistical power analyses across groups. Also, to elucidate the potential mechanisms for these six taxa, an analysis including metabolites would be necessary, but we could not conduct this analysis as metabolite data was not available in our study.

In conclusion, the identification of *Faecalibacterium prausnitzii*, *Prevotella copri*, unclassified *Dorea*, unclassified *Lachnospiraceae*, unclassified *Ruminococcus*, and unclassified *Sutterella* as potential modulators of IBD opens up exciting avenues for targeted therapeutic approaches. However, careful consideration of individual variations and further research is necessary to translate these findings into effective and personalized treatments for individuals with IBD.

## Conclusions

In this study, we showed that dysbiosis of the microbial community has different levels of impact on the inflammatory and immune response pathways by investigating the differences in the microbial community between healthy adolescents and those with GI diseases.

## Methods

### 16S rRNA-seq data collection from adolescents and their pre-processing

Inclusion Criteria: We conducted a keyword search for ‘adolescent microbiome’ on PubMed to identify datasets. We accessed datasets from the Sequence Retrieval Archive (SRA) [65] and Clinical and Omics Data Archive (https://coda.nih.go.kr). Our search yielded eight datasets (SRA accessions SRP035344, SRP058774, SRP064354, SRP082331, SRP114847, SRP126775, SRP173959; and CODA accession R000635) [66–75], totaling 434 samples. According to the publications for the datasets, informed consent was obtained, and adolescents were defined as individuals aged 10–19 [21]. They extracted DNA and performed sequencing of 16S rRNA gene from the DNA.

Exclusion Criteria: Some of the collected datasets included samples from adults or lacked information on disease/healthy control status. To address this, we excluded such samples, resulting in a total of 202 adolescents for the 16S rRNA-seq data analysis of the UC, CD, Ob, and HC groups for our study (Supplementary Table S3).

### OTUs and diversity analysis for investigating the differences in taxonomy proportions in each group

For the 202 selected samples from adolescents (HC, *n* = 92; Ob, n = 68; CD, *n* = 37; UC, *n* = 5), we used the fastx toolkit with a quality score > 25 and length 250 bp for trimming. We used Usearch (version. 61) [76] and QIIME [18] to remove chimera sequences and obtain the filtered fasta file. QIIME is a tool that processes the sequence data, including quality control, filtering, removal of chimeric sequences, and taxonomic classification of the microbes [18].

Using processed sequence data as input, QIIME [18] was used to analyze the gut microbiota taxonomy for each sample; we generated OTUs representing each taxon. To generate OTUs, we used greengenes (version 13_8) as a reference database [77] and the similarity was set at 99%. Subsample depth was set as 3000 for diversity analysis. Based on the OTU table, PCoA was performed using ‘phyloseq’ and ‘microbiome’ package in R (Version 4.0.2). A paired comparison was performed for each group (UC, CD, Ob, and HC), which was presented as a 2D PCoA plot to confirm clustering between the groups.

### Statistical significance of differences in taxonomy abundance and analysis of the associated KO terms and pathways

We used the PICRUSt tool to identify the taxa showing differences in proportions (i.e., OTU abundances) among the groups and to calculate ‘functional profiling’ (i.e., contributions of taxa to KO terms) [19]. PICRUSt is a tool that predicts functional profiling of microbial communities by taking the microbial abundance table (i.e. OTU) generated by QIIME [19] as input. Out of all taxa in the greengenes database [77], 55 taxa that showed significant differences in proportions among the groups were obtained under false discovery rate (FDR < 0.05). Further, we narrowed them down to six taxa such that each taxa showed a mean proportion of ≥ 5% in the group with the highest mean proportion. The STAMP [20] were used for visualization. STAMP is a tool that conducts statistical hypothesis testing for differences among the microbial metagenomic profiles for pairs of samples or groups of samples by for the differences and that visualizes the results from PICRUSt [20].” Given a taxa level, we compared different species (or phylum) by using proportions, and the comparisons were performed by ANOVA in STAMP [20].

### Pair-wise differences in contributions of the selected taxa to KO terms and pathways related to the selected taxa

Inspecting the functional profiling result from the PICRUSt, we obtained KO terms associated with the six selected taxa. Also, from the profiling result, we obtained the contribution of the six selected taxa to each KO term in a group. Then, given the KO term, we performed statistical tests using Tukey HSD to calculate pair-wise differences of the contribution of the selected taxa to the term among groups. Mapping the KO terms of the selected taxa to KEGG pathways were obtained from the functional profiling result from the PICRUSt.

### Supplementary Information

Additional file 1: Supplementary Figures S1 and S2; Supplementary Tables S1, S2 and S3.

## Data Availability

Datasets analyzed in this study were a re-analysis of existing data, which are openly available at Sequence Read Archive (SRA; https://www.ncbi.nlm.nih.gov/sra; accession numbers SRP035344, SRP058774, SRP064354, SRP082331, SRP114847, SRP126775) and Clinical & Omics Data Archive (CODA; https://coda.nih.go.kr; accession R000635).

## Abbreviations

2D PCoA: 2-Dimensional PCoA
UC: Ulcerative colitis
CD: Crohn’s disease
GI: Gastrointestinal
IBD: Inflammatory bowel disease
Ig: Immunoglobulin
KEGG: Kyoto Encyclopedia of Genes and Genomes
KO: KEGG orthology
Ob: Obesity
OTUs: Operational taxonomy units
PCoA: Principal coordinate analysis
PICRUSt: Phylogenetic Investigation of Communities by Reconstruction of Unobserved States
SCFAs: Short-chain fatty acids
PAMPs: Pathogen-associated molecular patterns
DAMPs: Damage-associated molecular patterns
PRRs: Pattern recognition receptors
QIIME: Quantitative Insights Into Microbial Ecology
STAMP: STatistical Analyses of Metagenomic Profiles
Tukey HSD: Tukey Honestly Significant Difference
