# Supplementary material for: Adolescent gut microbiome imbalance and its association with immune response in inflammatory bowel diseases and obesity
Source: BMC Microbiol. 2024 Jul 19;24:268. doi: 10.1186/s12866-024-03425-y (PMC11264842; doi:10.1186/s12866-024-03425-y)
Supplement: Supplementary file 1 — Additional file 1: Supplementary Figures S1 and S2; Supplementary Tables S1, S2 and S3. [file 12866_2024_3425_MOESM1_ESM.pdf]

Adolescent Gut Microbiome Imbalance and Its Association with Immune Response in Inflammatory Bowel Diseases and Obesity

Joo and Nam

Supplementary Figures S1 through S2
Supplementary Tables S1 through S3

Supplementary Figure S1. Beta-diversity PCoA plot of all groups

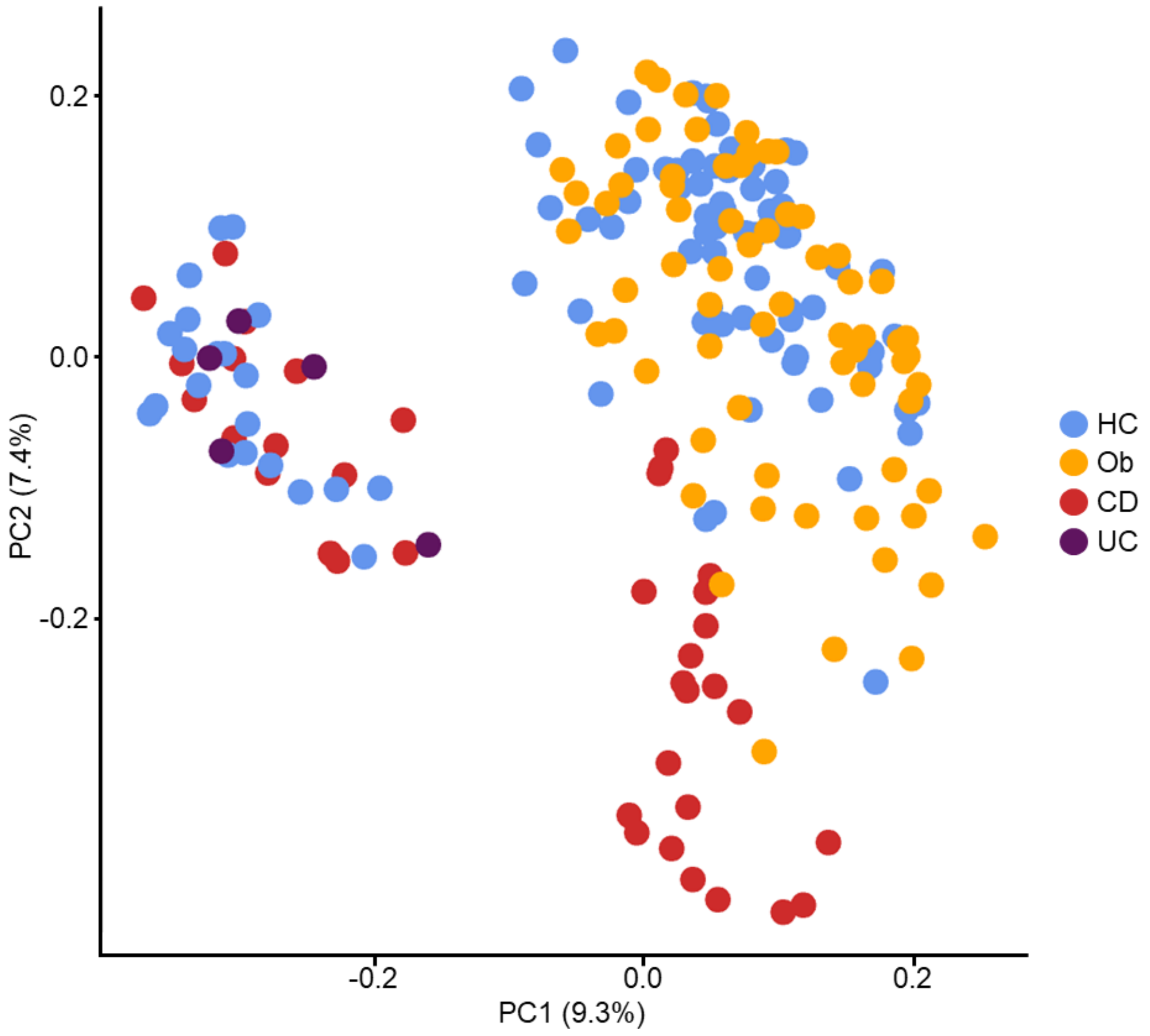

Supplementary Figure S2. Pair-wise comparisons of mean proportions in (A) *Dorea formicigenerans* and (B) *Dorea longicatena.*

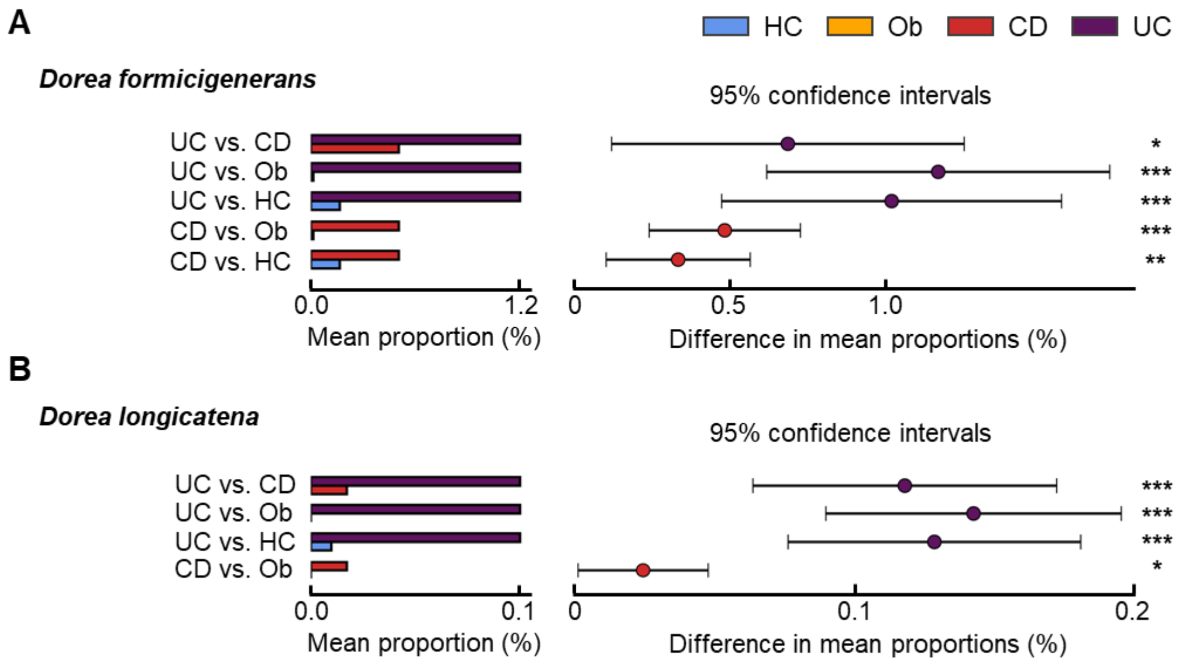

Supplementary Table S1. List of taxa showing statistically significant differences between groups through QIIME

Kingdom	Phylum	Class	Order	Family	Genus	Species	FDR
Bacteria	Acidobacteria	OS-K	Unclassified OS-K	Unclassified OS-K	Unclassified OS-K	Unclassified OS-K	1.03E-07
Bacteria	Actinobacteria	Actinobacteria	Actinomycetales	Brevibacteriaceae	Brevibacterium	casei	2.06E-07
Bacteria	Actinobacteria	Actinobacteria	Actinomycetales	Corynebacteriaceae	Corynebacterium	Unclassified Corynebacterium	0.0356
Bacteria	Actinobacteria	Actinobacteria	Actinomycetales	Nocardiaceae	Rhodococcus	fascians	1.65E-07
Bacteria	Actinobacteria	Actinobacteria	Actinomycetales	Promicromonosporaceae	Promicromonospora	Unclassified Promicromonospora	2.35E-07
Bacteria	Actinobacteria	Actinobacteria	Actinomycetales	Propionibacteriaceae	Propionibacterium	Unclassified Propionibacterium	5E-04
Bacteria	Actinobacteria	Coriobacteriia	Coriobacteriales	Coriobacteriaceae	Adlercreutzia	Unclassified Adlercreutzia	4.99E-10
Bacteria	Actinobacteria	Coriobacteriia	Coriobacteriales	Coriobacteriaceae	Collinsella	aerofaciens	0.023
Bacteria	Aquificae	Aquificae	Aquificales	Aquificaceae	Hydrogenobacter	subterraneus	1.50E-07
Bacteria	Bacteroidota	Bacteroidia	Bacteroidales	Odoribacteraceae	Odoribacter	Unclassified Odoribacter	0.007
Bacteria	Bacteroidota	Bacteroidia	Bacteroidales	Bacteroidaceae	Unclassified Bacteroidaceae	Unclassified Bacteroidaceae	1.37E-07
Bacteria	Bacteroidota	Bacteroidia	Bacteroidales	Prevotellaceae	Prevotella	copri	1.43E-07
Bacteria	Bacteroidota	Bacteroidia	Bacteroidales	Rikenellaceae	Alistipes	putredinis	0.002
Bacteria	Bacteroidota	Flavobacteriia	Flavobacteriales	Weeksellaceae	Cloacibacterium	Unclassified Cloacibacterium	0.003
Bacteria	Chloroflexi	C0119	Unclassified C0119	Unclassified C0119	Unclassified C0119	Unclassified C0119	1.10E-07
Bacteria	Firmicutes	Bacilli	Bacillales	Staphylococcaceae	Staphylococcus	haemolyticus	1.08E-05
Bacteria	Firmicutes	Bacilli	Gemellales	Gemellaceae	Gemella	Unclassified Gemella	0.049
Bacteria	Firmicutes	Bacilli	Lactobacillales	Carnobacteriaceae	Granulicatella	Unclassified Granulicatella	0.003
Bacteria	Firmicutes	Bacilli	Lactobacillales	Streptococcaceae	Streptococcus	anginosus	0.032
Bacteria	Firmicutes	Clostridia	Clostridiales	Mogibacteriaceae	Unclassified Mogibacteriaceae	Unclassified Mogibacteriaceae	0.018
Bacteria	Firmicutes	Clostridia	Clostridiales	Lachnospiraceae	Ruminococcus	gnavus	4.55E-05
Bacteria	Firmicutes	Clostridia	Clostridiales	Lachnospiraceae	Ruminococcus	Unclassified Ruminococcus	0.003

Bacteria	Firmicutes	Clostridia	Clostridiales	Lachnospiraceae	Anaerostipes	Unclassified Anaerostipes	0.007
Bacteria	Firmicutes	Clostridia	Clostridiales	Lachnospiraceae	Blautia	producta	1.09E-05
Bacteria	Firmicutes	Clostridia	Clostridiales	Lachnospiraceae	Blautia	Unclassified Blautia	0.012
Bacteria	Firmicutes	Clostridia	Clostridiales	Lachnospiraceae	Clostridium_dup1	citroniae	0.026
Bacteria	Firmicutes	Clostridia	Clostridiales	Lachnospiraceae	Dorea	Unclassified Dorea	8.46E-10
Bacteria	Firmicutes	Clostridia	Clostridiales	Lachnospiraceae	Dorea	longicatena	1.35E-07
Bacteria	Firmicutes	Clostridia	Clostridiales	Lachnospiraceae	Dorea	formicigenerans	1.93E-07
Bacteria	Firmicutes	Clostridia	Clostridiales	Lachnospiraceae	Roseburia	inulinivorans	0.044
Bacteria	Firmicutes	Clostridia	Clostridiales	Lachnospiraceae	Unclassified Lachnospiraceae	Unclassified Lachnospiraceae	4.06E-04
Bacteria	Firmicutes	Clostridia	Clostridiales	Oscillospiraceae	Faecalibacterium	prausnitzii	0.033
Bacteria	Firmicutes	Clostridia	Clostridiales	Oscillospiraceae	Oscillospira	Unclassified Oscillospira	6.88E-05
Bacteria	Firmicutes	Clostridia	Clostridiales	Unclassified Clostridiales	Unclassified Clostridiales	Unclassified Clostridiales	0.004
Bacteria	Firmicutes	Erysipelotrichi	Erysipelotrichales	Erysipelotrichaceae	Clostridium_dup2	ramosum_dup1	0.002
Bacteria	Fusobacteria	Fusobacteriia	Fusobacteriales	Fusobacteriaceae	Cetobacterium	somerae	9.74E-08
Bacteria	Pseudomonadota	Alphaproteobacteria	Rhizobiales	Methylobacteriaceae	Methylobacterium	adhaesivum	2.47E-07
Bacteria	Pseudomonadota	Alphaproteobacteria	Rhizobiales	Methylobacteriaceae	Unclassified Methylobacteriaceae	Unclassified Methylobacteriaceae	7.93E-06
Bacteria	Pseudomonadota	Alphaproteobacteria	Rhizobiales	Phyllobacteriaceae	Mesorhizobium	Unclassified Mesorhizobium	0.003
Bacteria	Pseudomonadota	Alphaproteobacteria	Rhodobacterales	Rhodobacteraceae	Sulfitobacter	guttiformis	1.27E-07
Bacteria	Pseudomonadota	Alphaproteobacteria	Rhodobacterales	Rhodobacteraceae	Thalassobacter	stenotrophicus	1.18E-07
Bacteria	Pseudomonadota	Betaproteobacteria	Burkholderiales	Alcaligenaceae	Sutterella	Unclassified Sutterella	0.003
Bacteria	Pseudomonadota	Deltaproteobacteria	Desulfobacterales	Desulfobulbaceae	Unclassified Desulfobulbaceae	Unclassified Desulfobulbaceae	4.54E-07
Bacteria	Pseudomonadota	Deltaproteobacteria	Desulfuromonadales	Desulfuromonadaceae	Unclassified Desulfuromonadaceae	Unclassified Desulfuromonadaceae	0.023
Bacteria	Pseudomonadota	Epsilonproteobacteria	Campylobacterales	Campylobacteraceae	Campylobacter	Unclassified Campylobacter	0.021
Bacteria	Pseudomonadota	Gammaproteobacteria	Alteromonadales	Alteromonadaceae	Cellvibrio	Unclassified Cellvibrio	3.29E-07

Bacteria	Pseudomonadota	Gammaproteobacteria	Alteromonadales	Shewanellaceae	Shewanella	Unclassified Shewanella	0.001
Bacteria	Pseudomonadota	Gammaproteobacteria	Enterobacteriales	Enterobacteriaceae	Enterobacter	pyrinus	4.27E-06
Bacteria	Pseudomonadota	Gammaproteobacteria	Enterobacteriales	Enterobacteriaceae	Plesiomonas	shigelloides	2.74E-07
Bacteria	Pseudomonadota	Gammaproteobacteria	Enterobacteriales	Enterobacteriaceae	Serratia	Unclassified Serratia	0.015
Bacteria	Pseudomonadota	Gammaproteobacteria	Pasteurellales	Pasteurellaceae	Aggregatibacter	pneumotropica	1.83E-07
Bacteria	Pseudomonadota	Gammaproteobacteria	Pasteurellales	Pasteurellaceae	Aggregatibacter	segnis	0.012
Bacteria	Pseudomonadota	Gammaproteobacteria	Pasteurellales	Pasteurellaceae	Aggregatibacter	Unclassified Aggregatibacter	0.049
Bacteria	Pseudomonadota	Gammaproteobacteria	Pseudomonadales	Moraxellaceae	Unclassified Moraxellaceae	Unclassified Moraxellaceae	0.023
Bacteria	SAR406	AB16	Arctic96B-7	A714017	ZA3312c	Unclassified ZA3312c	5E-04

Supplementary Table S2. List of 76 pathways (at the level 3) significantly different between groups through PICRUSt

Level_1	Level_2	Level_3	FDR
Metabolism	Amino Acid Metabolism	Alanine, aspartate and glutamate metabolism	3.02E-05
Metabolism	Amino Acid Metabolism	Cysteine and methionine metabolism	1.15E-05
Metabolism	Amino Acid Metabolism	Glycine, serine and threonine metabolism	1.82E-06
Metabolism	Amino Acid Metabolism	Lysine biosynthesis	6.48E-07
Metabolism	Amino Acid Metabolism	Phenylalanine, tyrosine and tryptophan biosynthesis	0.000275664
Metabolism	Amino Acid Metabolism	Valine, leucine and isoleucine biosynthesis	1.20E-09
Metabolism	Amino Acid Metabolism	Valine, leucine and isoleucine degradation	0.036046883
Metabolism	Biosynthesis of Other Secondary Metabolites	beta-Lactam resistance	2.13E-05
Metabolism	Biosynthesis of Other Secondary Metabolites	Streptomycin biosynthesis	3.17E-05
Metabolism	Carbohydrate Metabolism	Amino sugar and nucleotide sugar metabolism	8.79E-05
Metabolism	Carbohydrate Metabolism	Fructose and mannose metabolism	0.000261888
Metabolism	Carbohydrate Metabolism	Galactose metabolism	0.000122758
Metabolism	Carbohydrate Metabolism	Glycolysis / Gluconeogenesis	6.20E-11
Metabolism	Carbohydrate Metabolism	Glyoxylate and dicarboxylate metabolism	2.09E-06
Metabolism	Carbohydrate Metabolism	Pentose and glucuronate interconversions	5.16E-05
Metabolism	Carbohydrate Metabolism	Pentose phosphate pathway	3.45E-08
Metabolism	Carbohydrate Metabolism	Pyruvate metabolism	1.17E-11
Metabolism	Carbohydrate Metabolism	Starch and sucrose metabolism	9.75E-06
Metabolism	Energy Metabolism	Carbon fixation in photosynthetic organisms	1.67E-07
Metabolism	Energy Metabolism	Carbon fixation pathways in prokaryotes	5.96E-14
Metabolism	Energy Metabolism	Methane metabolism	4.79E-08
Metabolism	Energy Metabolism	Oxidative phosphorylation	3.72E-10
Metabolism	Energy Metabolism	Photosynthesis	1.89E-06
Metabolism	Glycan Biosynthesis and Metabolism	Peptidoglycan biosynthesis	3.81E-06

Metabolism	Lipid Metabolism	Fatty acid biosynthesis	0.00031964
Metabolism	Lipid Metabolism	Fatty acid metabolism	3.58E-07
Metabolism	Lipid Metabolism	Glycerolipid metabolism	1.06E-12
Metabolism	Lipid Metabolism	Glycerophospholipid metabolism	3.74E-09
Metabolism	Metabolism of Cofactors and Vitamins	Biotin metabolism	0.020567848
Metabolism	Metabolism of Cofactors and Vitamins	Folate biosynthesis	1.40E-10
Metabolism	Metabolism of Cofactors and Vitamins	Nicotinate and nicotinamide metabolism	1.37E-10
Metabolism	Metabolism of Cofactors and Vitamins	One carbon pool by folate	5.62E-11
Metabolism	Metabolism of Cofactors and Vitamins	Pantothenate and CoA biosynthesis	2.74E-06
Metabolism	Metabolism of Cofactors and Vitamins	Riboflavin metabolism	9.00E-13
Metabolism	Metabolism of Cofactors and Vitamins	Thiamine metabolism	6.00E-05
Metabolism	Metabolism of Cofactors and Vitamins	Vitamin B6 metabolism	1.09E-12
Metabolism	Metabolism of Other Amino Acids	Cyanoamino acid metabolism	0.001322133
Metabolism	Metabolism of Terpenoids and Polyketides	Biosynthesis of ansamycins	0.000179623
Metabolism	Metabolism of Terpenoids and Polyketides	Biosynthesis of vancomycin group antibiotics	3.88E-08
Metabolism	Metabolism of Terpenoids and Polyketides	Polyketide sugar unit biosynthesis	0.000173486
Metabolism	Metabolism of Terpenoids and Polyketides	Terpenoid backbone biosynthesis	2.45E-09
Metabolism	Metabolism of Terpenoids and Polyketides	Zeatin biosynthesis	2.04E-12
Metabolism	Nucleotide Metabolism	Purine metabolism	9.78E-11
Metabolism	Nucleotide Metabolism	Pyrimidine metabolism	5.15E-10
Metabolism	Xenobiotics Biodegradation and Metabolism	Drug metabolism - other enzymes	6.35E-10
Human Diseases	Cancers	Pathways in cancer	6.22E-09
Human Diseases	Cancers	Prostate cancer	2.02E-05
Human Diseases	Infectious Diseases	Epithelial cell signaling in Helicobacter pylori infection	7.03E-06
Human Diseases	Infectious Diseases	Tuberculosis	5.75E-07
Human Diseases	Metabolic Diseases	Type I diabetes mellitus	1.91E-05

Cellular Processes	Cell Growth and Death	Cell cycle - Caulobacter	4.61E-09
Cellular Processes	Cell Motility	Flagellar assembly	0.000899946
Cellular Processes	Transport and Catabolism	Peroxisome	0.000243213
Organismal Systems	Endocrine System	Adipocytokine signaling pathway	1.12E-10
Organismal Systems	Endocrine System	PPAR signaling pathway	0.000130035
Organismal Systems	Endocrine System	Progesterone-mediated oocyte maturation	2.87E-07
Organismal Systems	Environmental Adaptation	Plant-pathogen interaction	0.003488418
Organismal Systems	Immune System	Antigen processing and presentation	2.90E-07
Organismal Systems	Immune System	NOD-like receptor signaling pathway	0.000156488
Organismal Systems	Nervous System	Glutamatergic synapse	0.011546008
Genetic Information Processing	Folding, Sorting and Degradation	Protein export	3.02E-09
Genetic Information Processing	Folding, Sorting and Degradation	Protein processing in endoplasmic reticulum	7.60E-10
Genetic Information Processing	Folding, Sorting and Degradation	RNA degradation	1.49E-12
Genetic Information Processing	Folding, Sorting and Degradation	Sulfur relay system	1.94E-13
Genetic Information Processing	Replication and Repair	Base excision repair	0.000110797
Genetic Information Processing	Replication and Repair	DNA replication	3.06E-10
Genetic Information Processing	Replication and Repair	Homologous recombination	1.07E-11
Genetic Information Processing	Replication and Repair	Mismatch repair	5.15E-09
Genetic Information Processing	Replication and Repair	Nucleotide excision repair	3.31E-08
Genetic Information Processing	Transcription	RNA polymerase	9.36E-05
Genetic Information Processing	Translation	Aminoacyl-tRNA biosynthesis	2.09E-06
Genetic Information Processing	Translation	Ribosome	1.12E-09
Genetic Information Processing	Translation	Ribosome biogenesis in eukaryotes	7.29E-09
Environmental Information Processing	Membrane Transport	ABC transporters	2.24E-10
Environmental Information Processing	Membrane Transport	Bacterial secretion system	0.006754005
Environmental Information Processing	Signal Transduction	Two-component system	2.75E-07

Supplementary Table S3. Description of the 16S rRNA-seq datasets.

Dataset	# of control samples	# of experimental samples	Country	Publication (year)	Informed consent	Included in our study?
SRP035344	4 (Healthy)	0	China	PMID: 26443005 (2015) [8]	Yes	Yes
SRP058774	21 (Healthy)	20 (UC, CD)	USA	PMID: 27006956 (2016) [10]	Not available	Yes
SRP064354	0	22 (CD)	USA	PMID: 26804920 (2016) [9]	Yes	Yes
SRP082331	-	-	USA	PMID: 27648960 (2016) [11]	Yes	No (healthy/IBD status unknown)
SRP114847	-	-	India	PMID: 30698687 (2019) [14]	Yes	No (healthy/IBD status unknown)
SRP126775	-	-	USA	PMID: 29493105 (2018) [13]	Yes	No (healthy/IBD status unknown)
SRP173959	-	-	Kenya	PMID: 31015324 (2019) [15]	Not available	No (healthy/IBD status unknown)
CODA R000635	67 (Healthy)	68 (Obese)	Korea	PMID: 28676106 (2017) [12]	Not available	Yes

"-": In the dataset, there were no samples of healthy adolescents, and there were no adolescents with IBD (UC and CD) or obese adolescents.
